# The Impact of Near-Infrared Spectroscopy in Early Detection of Cerebral Deterioration After Aneurysmal Subarachnoid Haemorrhage

**DOI:** 10.3390/jcm15041349

**Published:** 2026-02-09

**Authors:** Ieva Būce-Šatoba, Gaida Krūmiņa, Agnese Ozoliņa

**Affiliations:** 1 Department of Anaesthesiology, Intensive Care and Clinical Simulation, Riga Stradiņš University, LV-1007 Riga, Latvia; 2 Riga East University Hospital, 2 Hipokrata Str., LV-1079 Riga, Latvia; 3 Department of Radiology, Riga Stradiņš University, LV-1007 Riga, Latvia

**Keywords:** near-infrared spectroscopy, aneurysmal subarachnoid haemorrhage, delayed cerebral ischemia, cerebral vasospasm, regional cerebral oxygen saturation, neuromonitoring, intensive care

## Abstract

**Background/Objectives**: Delayed cerebral ischemia (DCI) represents a major cause of morbidity and mortality after aneurysmal subarachnoid haemorrhage (aSAH). Early identification of developing cerebral ischemia is essential for timely prevention of DCI. Near-infrared spectroscopy (NIRS) provides continuous, non-invasive bedside monitoring of regional cerebral oxygen saturation (rSO_2_); however, its clinical value in patients with aSAH has not yet been fully established. The primary objective of this study was to investigate whether NIRS-detected rSO_2_ desaturation can serve as an early indicator of cerebral vasospasm (CV) and predict the occurrence of DCI. Secondary objectives were to examine the associations between rSO_2_ changes and other cerebral deterioration events, length of intensive care unit stay, functional outcome, and in-hospital mortality. **Methods**: This prospective, single-centre study included 30 patients with aSAH admitted to the intensive care unit (ICU) of Riga East University Hospital between January 2019 and January 2023. Bilateral frontal near-infrared spectroscopy (NIRS) monitoring (Covidien INVOS™ 5100C-PB) was initiated within 72 h after ictus and continued for up to 7 days. Cerebral desaturation was defined as a >20% reduction from baseline (BL) or an absolute regional cerebral oxygen saturation (rSO_2_) value < 50% lasting ≥30 min. CV and DCI were diagnosed according to established clinical and radiological criteria. Receiver operating characteristic (ROC) analysis was performed to evaluate the sensitivity and specificity of rSO_2_ thresholds for the detection of CV, DCI, and other cerebral deterioration events. **Results**: CV occurred in 10 patients (33%); however, only four cases were detected during the NIRS monitoring period. NIRS demonstrated very high sensitivity (97.5%) but extremely low specificity (6%) for the early detection of CV. In contrast, diagnostic accuracy for DCI was high. An absolute rSO_2_ cut-off value of 52% yielded a sensitivity of 97.5% and a specificity of 95%, whereas a decrease of ≥26% from baseline (BL) demonstrated a sensitivity of 98% and a specificity of 93%. Significant rSO_2_ reductions were also observed during aneurysm re-rupture, hydrocephalus, cerebral edema, and postoperative ischemia; however, the sensitivity of NIRS for detecting these events was negligible. Patients with ≥20% desaturation tended to have longer ICU stays, and lower mean rSO_2_ values as well as greater desaturation were associated with poorer functional outcomes as assessed by the modified Rankin Scale. Patients who died exhibited more pronounced rSO_2_ decreases and less recovery compared with survivors. **Conclusions**: In this cohort, NIRS demonstrated limited specificity for the early detection of CV but showed strong associations with DCI and neurological outcome. NIRS may be useful as a non-invasive adjunct to multimodal neuromonitoring rather than as a stand-alone diagnostic tool for cerebral vasospasm. Larger, prospective studies incorporating standardized imaging protocols and optimized rSO_2_ thresholds are required to more clearly define the role of NIRS in the management of aSAH.

## 1. Introduction

Despite survival after the initial cerebral aneurysm rupture, patients remain at risk of developing secondary complications that may lead to delayed cerebral ischemia (DCI), poor neurological outcome, or death [1]. Severe complications include cerebral edema, cortical spreading depolarization, perfusion mismatch, microthrombosis, blood–brain barrier disruption, oxidative stress, and cerebral vasospasm (CV) [1]. Cerebral vasospasm occurs in approximately 30% of patients with aneurysmal subarachnoid haemorrhage (aSAH). Typically, CV develops several days after aSAH, most often within 3–14 days, with peak incidence in the first week after ictus and is associated with an increased risk of progression to DCI [2,3,4,5]. Therefore, early detection of evolving cerebral ischemia is crucial before irreversible injury occurs.

According to a definition proposed in 2010 by a multidisciplinary research group, DCI comprises two main components: a clinical component, defined as the development of new focal neurological deficits or a decrease in the level of consciousness not attributable to other causes, and a radiological component, defined by the presence of cerebral infarction on imaging [6].

It should be emphasized that a detailed neurological examination remains the most reliable tool for monitoring and detecting DCI [7]. However, in comatose patients, clinical assessment is inherently limited, making the diagnosis of DCI particularly challenging.

Transcranial Doppler (TCD) ultrasound monitoring is recommended in the latest American Heart Association/American Stroke Association (AHA/ASA) guidelines for the management of subarachnoid haemorrhage (2023) for follow-up, early detection CV, and prediction of DCI [8]. However, TCD has several important limitations. It primarily detects macrovascular vasospasm and is unable to assess vasospasm in distal or peripheral vessels. In addition, the method is operator-dependent, does not provide continuous monitoring, and is typically performed only once or twice daily. TCD measurements may also be limited by individual patient anatomy [6,8]. Furthermore, growing evidence suggests that CV alone does not fully explain the development of DCI [9].

Near-infrared spectroscopy (NIRS) is a non-invasive bedside monitoring technique that provides continuous, real-time measurement of regional cerebral oxygen saturation (rSO_2_), reflecting the balance between cerebral oxygen supply and consumption within the monitored region [10]. A comparative study by Brawanski et al. demonstrated a significant correlation between invasive brain tissue oxygen monitoring and rSO_2_ in patients with aSAH and traumatic brain injury [11]. Several studies have suggested that NIRS may be a useful monitoring modality for improving the prediction of DCI and clinical outcomes following aSAH [12,13,14,15,16,17].

Nevertheless, to date, available data have shown conflicting results regarding the diagnostic value of NIRS for predicting DCI in patients with SAH [8]. A concise review by Francoeur et al. (2022) concluded that current evidence does not support the use of NIRS to guide the clinical management of patients with aSAH [10].

Invasive neuromonitoring (INM) is also used as a continuous bedside method for monitoring brain oxygenation and cerebral metabolic status, providing real-time data with good predictive value for DCI. However, the clinical applicability of invasive neuromonitoring is limited by the relatively high incidence of procedure-related complications, including periprocedural haemorrhage at the probe insertion site reported in up to 20% of cases, as well as by the restricted spatial representation of brain tissue being monitored. These limitations reduce the feasibility of routine use of invasive techniques in all patients with aSAH and highlight the need for reliable non-invasive monitoring approaches in future clinical practice [18].

Based on these considerations, we hypothesized that NIRS is a safe modality that can be successfully applied in patients with aSAH regardless of haemorrhage severity to enable timely detection of cerebral deterioration. Accordingly, the primary aim of this study was to evaluate the ability of NIRS-detected rSO_2_ desaturation to identify early CV and to predict DCI in patients with aSAH treated in the intensive care unit (ICU). Secondary aims were to assess the associations between rSO_2_ desaturation and other cerebral deterioration events, length of ICU stay and total hospital stay, functional outcome, and in-hospital mortality.

## 2. Materials and Methods

This prospective study was conducted in the ICU of Riga East University Hospital, Riga, Latvia, between January 2019 and January 2023. The study was performed in accordance with the principles of the Declaration of Helsinki and was approved by the Ethics Committee of Riga Stradiņš University (Riga, Latvia), approval No. 6-2/6/31; 20 August 2019, as well as by Riga East University Hospital (Riga, Latvia), approval No. ZD/08-06/01-19/253; 21 October 2019. Written informed consent was obtained from all patients or, in cases where patients were unable to provide consent, from their legally authorized representatives prior to study enrolment.

Artificial intelligence-based tools were not used for study design, data collection, data analysis, or interpretation of the results.

### 2.1. Patient Selection and Groups

All patients with aSAH who were admitted to the ICU and met the inclusion criteria were enrolled in the study. The inclusion criteria were age > 18 years; level of consciousness assessed by the Glasgow Coma Scale (GCS) score of 5–15; confirmed cerebral aneurysm rupture on computed tomography angiography (CTA); any extent of SAH according to the Fisher radiological classification (grades I–IV); and an interval of less than 72 h from ictus to study inclusion.

The exclusion criteria were SAH of other aetiologies (including spontaneous SAH without confirmed vascular pathology, rupture of an arteriovenous malformation, rupture of a dural arteriovenous fistula, or traumatic SAH), level of consciousness with a GCS score < 5, and an interval of more than 72 h from ictus.

### 2.2. Data Collection and Therapeutic Management

Patient demographic characteristics and medical history were obtained from the patients or their relatives, as well as from medical records. Information regarding the extent of aSAH and the location of ruptured aneurysm was collected based on non-contrast computed tomography (CT) and computed tomography angiography (CTA) of the head performed at admission to the emergency department.

Monitoring of regional cerebral oxygen saturation (rSO_2_) using near-infrared spectroscopy (NIRS) was initiated within the first 72 h after ictus in all patients with aneurysmal subarachnoid haemorrhage who met the inclusion criteria and were admitted to the intensive care unit. NIRS monitoring was performed using the Covidien INVOS™ 5100C-PB Cerebral/Somatic Oximeter (Medtronic, Watford, UK). Disposable sensors with an integrated near-infrared light source and photodetector were placed bilaterally on the forehead, 3 cm above the superciliary line, with the long axis oriented parallel to the interaural line, after cleansing the forehead skin with Skinsept^®^ pur (Ecolab Europe GmbH, Monheim am Rhein, Germany) [19]. Monitoring was continued for up to 7 days after ictus. According to the manufacturer’s recommendations, disposable sensors were replaced for each new patient. As the manufacturer specifies that sensors used for continuous monitoring do not require replacement unless they show signs of wear or become compromised, one set of sensors was used per patient in most cases, with daily inspection of sensor integrity [19,20]. The baseline (BL) rSO_2_ value was defined as the measurement obtained 5 min after initiation of NIRS monitoring.

NIRS data were updated every 5–6 s [20] and automatically recorded in the monitor case history at 30 s intervals. Trend graphs were reviewed at least once every 24 h, and mean rSO_2_ values were documented in the study protocol on an hourly basis.

All patients received enteral nimodipine (Nimotop^®^, 30 mg; Bayer AG, Berlin, Germany) at a dose of 60 mg every 6 h after admission to the ICU for the prevention of CV. If enteral administration was not feasible due to vomiting or gastric stasis, nimodipine (Nimotop^®^, 0.2 mg/mL; Bayer AG, Germany) was administered intravenously. In cases of diagnosed CV, nimodipine was administered intra-arterially. Intravenous magnesium sulfate (Magnesii sulfas heptahydricus, 250 mg/mL; Kalceks, Riga, Latvia) was administered to correct hypomagnesaemia. Vasopressor support with norepinephrine (Noradrenalinum, 1 mg/mL; Fresenius Kabi, Wroclaw, Poland) was administered as needed to maintain a mean arterial pressure between 70 and 100 mmHg.

Any new radiological findings and changes in the patient’s clinical status were prospectively recorded in the study protocol.

### 2.3. Measured Outcomes

The primary outcome was the evaluation of the sensitivity and specificity of NIRS-detected regional cerebral oxygen saturation (rSO_2_) desaturation for the early recognition of CV and the prediction of DCI in patients with aneurysmal subarachnoid haemorrhage.

Secondary outcomes included the assessment of the association between rSO_2_ desaturation and other cerebral deterioration events. In addition, we evaluated the relationships between early-detected cerebral deterioration and length of ICU stay, total hospital stays, functional outcome at discharge, and in-hospital mortality.

### 2.4. Definitions

*Cerebral deterioration* was defined as the occurrence of a new focal neurological deficit or a decrease of at least 2 points on the GCS lasting for more than 1 h, not occurring immediately after aneurysm occlusion, and not attributable to other causes [7].

*Cerebral desaturation* was defined as a decrease in rSO_2_ of > 20% from baseline (BL) or an absolute rSO_2_ value <50% lasting for at least 30 min [19,21,22].

The *cerebral deterioration due to other events* was defined as cerebral desaturation not caused by CV but associated with cerebral edema, new acute intracranial haemorrhage, hydrocephalus, or postoperative ischemia.

*Cerebral infarction* was defined as the presence of cerebral infarction on brain CT or magnetic resonance (MR) imaging within 6 weeks after subarachnoid haemorrhage, not present on CT or MR imaging performed between 24 and 48 h after early aneurysm occlusion, and not attributable to other causes such as surgical clipping or endovascular treatment [7].

*Cerebral vasospasm (CV)* was diagnosed and confirmed by computed tomography angiography (CTA) or digital subtraction angiography (DSA).

*Delayed cerebral ischemia (DCI)* was defined as the presence of cerebral infarction on brain CT does not present between 24 and 48 h after early aneurysm occlusion and not attributable to other causes such as surgical clipping or endovascular treatment [7].

*Length of stay in ICU* was defined as the total number of days spent in the ICU from the time of ICU admission until death or transfer to the neurosurgery department.

*Total hospital stay* was defined as the total number of days spent in the hospital from admission until death, discharge home, or transfer to a lower-level hospital.

*Functional outcome at discharge* was assessed using the modified Rankin Scale (mRS) on the day of hospital discharge. Functional status was categorized as follows [23]:

mRS 0—no symptoms;
mRS 1—no significant disability;
mRS 2—slight disability;
mRS 3—moderate disability;
mRS 4—moderately severe disability;
mRS 5—severe disability;
mRS 6—death.

*In-hospital mortality* was defined as death occurring at any time during the hospital stay.

### 2.5. Statistical Analysis

Data were analyzed using SPSS software (IBM SPSS Statistics, version 27; IBM Corp., Armonk, NY, USA). Continuous variables are presented as mean ± standard deviation (SD), and categorical variables as percentages (%). Comparisons between patient groups were performed using the Mann–Whitney U test for non-parametric variables and the independent-samples *t* test or analysis of variance (ANOVA) for parametric variables, as appropriate. The chi-square test was used to analyse categorical data. The Kruskal–Wallis test was used to compare continuous and ordinal variables across more than two independent groups when data did not meet the assumptions of normal distribution. Receiver operating characteristic (ROC) curve analysis was performed to evaluate the ability of NIRS to detect cerebral vasospasm, delayed cerebral ischemia, and other causes of cerebral deterioration, including calculation of the area under the curve (AUC) for each outcome. The Youden index was used to identify optimal rSO_2_ cut-off values by maximizing the sum of sensitivity and specificity. Statistical significance was defined as a *p* value < 0.05.

## 3. Results

### 3.1. Characteristics of aSAH Patients and Complications

A total of 30 patients with aSAH admitted to the ICU were included in this prospective study and monitored using NIRS for up to 7 days after ictus. Overall, isolated CV was detected in 6 patients (20%), isolated DCI in 7 patients (23%), and a combination of CV and DCI in 4 patients (14%), as shown in Table 1. CV was diagnosed during the NIRS monitoring period in only four cases, whereas in the remaining six patients it was identified later, up to day 14 after ictus.

According to the applied definitions, DCI developed because of CV in four patients. In seven patients, secondary cerebral ischemia was identified within 24–48 h after aneurysm occlusion and was attributed to intracranial haemorrhage due to aneurysm re-rupture or cerebral edema. In some cases, DCI occurred without a clearly identifiable underlying cause. Overall, various early and late complications of aSAH were observed in 26 patients (87%).

Detailed cohort characteristics stratified by the occurrence of CV, DCI, or both are presented in Table 1 for the entire study population. Most patients were female (63%), with a mean age of 62 ± 12 years and a mean body mass index of 27.3 ± 5.8 kg/m^2^. Patients who developed CV tended to be younger males with obesity. No other significant associations were identified between comorbidities and the development of CV or DCI. Additionally, CV occurred exclusively in patients with poor-grade aSAH or Fisher grade IV haemorrhage according to radiological classification, although this association did not reach statistical significance (*p* = 0.08).

In 19 of 30 patients (63%), the ruptured aneurysm was occluded within the first 48 h after admission, predominantly by endovascular embolization compared with neurosurgical clipping (60% vs. 37%) and in one case (3%) patient received a combination of both approaches.

The median length of stay in the ICU across all groups was 11 days (range, 3–43 days). Overall, in-hospital mortality was 30% (9 of 30 patients). Mortality was 33% (2 of 6 patients) in the CV group and was higher in the combined CV and DCI group, reaching 50% (2 of 4 patients).

### 3.2. Association Between NIRS-Detected rSO_2_ Desaturation, Cerebral Vasospasm, Delayed Cerebral Ischemia, and ICU Length of Stay

A total of 7262 rSO_2_ measurements obtained by NIRS were analyzed. Cerebral desaturation defined as an rSO_2_ decrease of >20% from baseline (BL) associated with CV was observed in 4 of 10 patients who exhibited rSO_2_ desaturation during the 7-day monitoring period. Among patients who subsequently developed DCI, significant rSO_2_ decreases were observed in 9 cases.

In the entire study population, the mean BL rSO_2_ value was 72 ± 8% on the left hemisphere and 72 ± 7% on the right hemisphere. BL rSO_2_ values ranged from 56% to 92%. No significant differences in BL rSO_2_ values were detected between patients who developed CV during the monitoring period and those who did not (73 ± 7% vs. 72 ± 9%, *p* = 0.83 on the left side; 71 ± 5% vs. 72 ± 8%, *p* = 0.64 on the right side).

**Figure 1:**
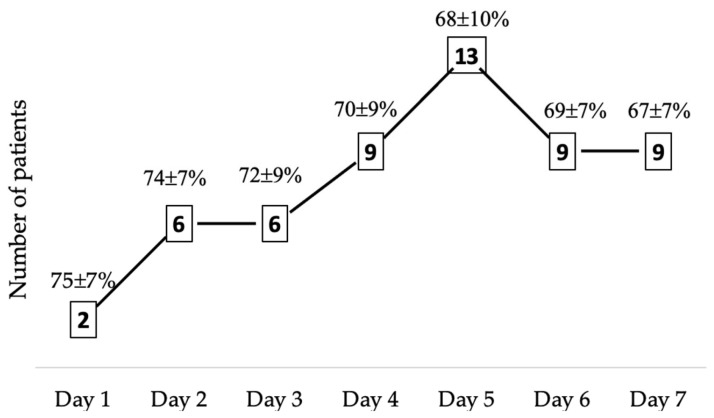
Number of patients exhibiting cerebral deoxygenation, defined as an rSO_2_ decrease of ≥20% from baseline.

The highest incidence of cerebral desaturation (>20% decrease from BL) was observed on day 5 after ictus (Figure 1), occurring in 13 patients (43%) and attributable to various underlying causes. The maximum observed desaturation reached a 59% reduction from baseline, and the lowest recorded absolute rSO_2_ value was 29%.

For the early detection of CV, NIRS demonstrated a high sensitivity of 97.5% but very low specificity of 6% at an rSO_2_ cut-off value of 54.5% (AUC = 0.41; 95% CI, 0.39–0.43; *p* < 0.05). In addition, a decrease in cerebral oxygenation with a cut-off value of a 12% reduction from BL showed a sensitivity of 91% and a specificity of 24% for the prediction of CV (AUC = 0.55; 95% CI, 0.536–0.568; *p* < 0.05).

For the detection of DCI, ROC curve analysis demonstrated a sensitivity of 97.5% and a specificity of 95% at an absolute rSO_2_ cut-off value of 52% (AUC = 0.48; CI, 0.473–0.5; *p* = 0.045). For the prediction of DCI, a decrease in rSO_2_ of ≥26% from BL yielded a sensitivity of 98% and a specificity of 93% (AUC = 0.50; 95% CI, 0.481–0.508; *p* = 0.50).

Patients who experienced cerebral desaturation of ≥20% from BL tended to have longer ICU stays compared with patients without a decrease in rSO_2_ (median, 13 vs. 8 days; *p* = 0.08). In addition, a weak but statistically significant negative correlation was observed between mean rSO_2_ values and length of stay in the ICU (*r* = −0.20, *p* < 0.005).

### 3.3. Changes in rSO_2_ Cerebral Desaturation Associated with Other Cerebral Deterioration Events

In addition to CV and DCI, other events associated with cerebral deterioration were observed in 26 patients. Significant reductions in rSO_2_ were recorded during aneurysm re-rupture occurring before or during aneurysm occlusion in 8 patients (27%), in cases of hydrocephalus in 7 patients (23%), cerebral edema in 5 patients (17%), and postoperative ischemia in 3 patients (10%).

In one case, cerebral desaturation was associated with an acute postoperative intracranial haemorrhage due to aneurysm re-rupture, while in three cases it was associated with cerebral edema. For these events, NIRS demonstrated very limited diagnostic performance, with negligible sensitivity and specificity, as reflected by a low area under the curve (AUC = 0.009; *p* < 0.05).

### 3.4. Relationship Between rSO_2_ Cerebral Desaturation, Functional Outcome, and Mortality

As shown in Table 2, mean rSO_2_ values were higher in patients without neurological deficits compared with those who experienced more severe neurological impairment, as assessed by the modified Rankin Scale (mRS). In addition, the maximal decrease in rSO_2_ from BL was greater in patients with poor neurological outcomes, particularly among those with an mRS score of 5.

Although patients who died had, on average, higher baseline rSO_2_ values compared with survivors (72 ± 8% vs. 70 ± 10%, *p* < 0.005), they experienced a greater decrease in rSO_2_ from BL and showed substantially less recovery over time, as presented in Table 3.

## 4. Discussion

None of the currently available non-invasive or invasive neuromonitoring modalities has strong guideline recommendations, and each is associated with specific advantages and limitations [6]. Despite advances in aneurysm repair techniques and critical care management strategies, aSAH remains associated with substantial morbidity and mortality, with reported mortality rates of up to 25% and disability rates of approximately 66%, particularly among patients with high-grade aSAH [24,25].

In this study, we aimed to evaluate the ability of NIRS-detected cerebral desaturation to identify early CV in patients with aSAH treated in the ICU, with the goal of preventing progression to DCI. In addition, we assessed the associations between rSO_2_ desaturation and other cerebral deterioration events, functional outcome, length of stay in the ICU, and in-hospital mortality.

Based on our results, NIRS appears to be a safe and feasible non-invasive neuromonitoring method for assessing rSO_2_. Although mean rSO_2_ values were lower in patients with neurological deficits and cerebral desaturation was associated with various cerebral deterioration events, NIRS demonstrated insufficient specificity for the early detection of CV. Nevertheless, changes in rSO_2_ values may have prognostic relevance. In the present study, lower mean rSO_2_ values and greater degrees of desaturation were associated with poorer functional outcomes as assessed by the modified Rankin Scale. Similarly, patients who experienced cerebral desaturation tended to have prolonged ICU stays compared with those without rSO_2_ decreases (median, 13 vs. 8 days; *p* = 0.08).

During the 7-day NIRS monitoring period, isolated CV was diagnosed in 6 patients (20%); however, in an additional 4 patients (13%), CV was identified later, up to day 14 after ictus, resulting in a total of 10 patients (33%) with CV. We hypothesize that in four cases CV may not have been detected during the monitoring period because radiological examinations were performed more than 24 h after the initial decrease in rSO_2_, at a time when DCI had already developed. Furthermore, CV occurred exclusively in patients with poor-grade aSAH. A similar trend has been reported by Samuels et al., who demonstrated that patients with high-grade aSAH have a higher risk of unfavourable outcomes despite revised treatment strategies, based on an analysis of 2475 patients [25].

Although a decrease in rSO_2_ was observed in all patients who developed cerebral vasospasm, NIRS demonstrated limited diagnostic performance for the prediction of cerebral vasospasm, characterized by high sensitivity but very low specificity (AUC = 0.41; 95% CI, 0.39–0.43; *p* < 0.05).

One possible explanation is that NIRS-derived rSO_2_ represents a composite signal of arterial and venous oxygenation, with the arterial component accounting for only approximately 25%. As a result, NIRS may not adequately capture changes in cerebral blood flow associated with vasospasm, which could explain its limited specificity and sensitivity for the early diagnosis of CV, contrary to our initial hypothesis [26]. Furthermore, a recent study by Leone et al. also reported limited utility of NIRS for predicting CV in patients with poor-grade aSAH, supporting our findings [27].

DCI, as defined by the established criteria, occurred in 7 patients (23%); however, secondary cerebral ischemia was identified in 11 patients (37%), of whom 10 exhibited cerebral deoxygenation exceeding 20% from BL (*p* = 0.06). In contrast to CV, ROC curve analysis demonstrated high sensitivity (97.5%) and specificity (95%) for the detection of DCI at an absolute rSO_2_ cut-off value of 52% (AUC = 0.48; CI, 0.473–0.5; *p* = 0.045). Similarly, rSO_2_ decreases of ≥26% from BL demonstrated high sensitivity (98%) and specificity (93%) for the prediction of DCI (AUC = 0.50; 95% CI, 0.481–0.508; *p* = 0.50). Because the precise onset of cerebral ischemia could not be determined, it remains unclear whether the observed cerebral deoxygenation preceded ischemic injury or occurred as a consequence of established ischemia.

The rSO_2_ threshold values in this study were derived using the Youden index from ROC curve analysis, allowing for an objective selection of cut-off points that balance sensitivity and specificity. Given the exploratory nature of the study and the limited sample size, these thresholds should be interpreted cautiously and validated in larger cohorts.

Comparable findings regarding DCI have been reported by other authors, demonstrating relatively high sensitivity and specificity of NIRS for the detection of DCI [12,13]. In a study by van der Harst et al. (2023) [12] including 41 patients with aSAH, DCI developed in 12 patients. Consistent with our results, the authors reported an optimal rSO_2_ cut-off value of 65% for predicting DCI, yielding a sensitivity of 100% and a specificity of 45% [12]. Similarly, Park et al. observed that an rSO_2_ decrease of at least 12.7% was associated with a sensitivity of 94.44% (95% CI: 72.7–99.9%) and a specificity of 70.59% (95% CI: 52.5–84.9%) for the detection of DCI [13]. Taken together, these findings raise the question of whether the predefined rSO_2_ reduction thresholds applied in the present study may have been overly stringent.

In addition, cerebral desaturation was observed not only in patients with established CV and DCI but also in other acute cerebral events. In one case, desaturation occurred in the context of acute postoperative intracranial haemorrhage, and in three cases it was associated with cerebral edema. Although a close temporal relationship was clinically observed between sudden decreases in rSO_2_ and acute intracerebral haemorrhage, NIRS demonstrated limited diagnostic performance for these events (AUC = 0.009; *p* < 0.05). Currently, data supporting the use of NIRS for the diagnosis of acute cerebral events other than cerebral ischemia remain limited. To date, several studies have evaluated portable NIRS devices as screening tools for traumatic intracranial haemorrhage, reporting promising results; however, these technologies differ substantially from the continuous cerebral oximetry systems used in the present study and are therefore not directly comparable [28,29,30].

As a secondary objective, we evaluated the impact of rSO_2_ cerebral desaturation on length of stay in the ICU and hospital, functional outcome, and mortality. Patients who experienced cerebral desaturation of ≥20% from BL tended to have longer ICU stays compared with those without desaturation (median, 13 vs. 8 days); however, this difference did not reach statistical significance. These findings suggest that cerebral desaturation may not be specific to the pathophysiology of aSAH. In support of this interpretation, studies in cardiac surgical populations have demonstrated that intraoperative cerebral desaturation is associated with an increased risk of neurological complications and prolonged ICU or hospital stay [31].

Nevertheless, injured brain tissue is considerably more sensitive and vulnerable to hypoxia than healthy brain tissue. From this perspective, it may be hypothesized that patients who experience reductions in rSO_2_ are more likely to have an unfavourable clinical course, requiring greater intensity of intensive care support, compared with patients who maintain preserved cerebral oxygenation.

Similarly, when analysing functional outcomes as assessed by the modified Rankin Scale, we observed an association between rSO_2_ values and neurological outcome. Patients with good neurological outcomes (mRS 0) exhibited the highest mean rSO_2_ values, whereas those with poor neurological outcomes (mRS 5) demonstrated more pronounced decreases in rSO_2_ from baseline. These findings are consistent with those reported by van der Harst et al., who showed that lower mean rSO_2_ values after subarachnoid haemorrhage were strongly associated with unfavourable functional outcomes (mRS > 3) [12]. Likewise, Rivera-Lara et al. reported that reductions in rSO_2_ correlated with poor neurological outcomes [32]. Finally, in-hospital mortality in our cohort reached 30%, which is slightly higher than the mortality rates reported in the literature. Among survivors, disability defined as an mRS score ≥ 3 was observed in 76% of patients at hospital discharge, consistent with previously reported outcomes in patients with aSAH [24].

This study has several limitations that should be acknowledged. The most important limitations include the small sample size and the absence of a standardized radiological imaging protocol. The limited cohort size reduces statistical power and limits the precision of diagnostic accuracy, while non-standardized imaging may have affected the timing of CV detection. The number of included patients was directly limited by the incidence of aSAH cases admitted to the emergency department during the predefined study period approved by the institutional ethics committees. Based on the observed incidence of CV in our cohort, a minimum sample size of approximately 210 patients would be required to achieve 80% statistical power. According to data from the Statistics Department of Riga East University Hospital (Riga, Latvia), between 39 and 114 patients with aneurysmal subarachnoid haemorrhage were admitted annually to the ICU during the study period, and not all these patients met the inclusion criteria. An additional major confounding factor was the COVID-19 pandemic, which substantially restricted patient monitoring and study procedures due to quarantine and infection control regulations. Furthermore, some patients remained in the intensive care unit for less than 7 days, which limited the duration of NIRS monitoring and resulted in incomplete data collection for certain cases. Future studies should aim to overcome these limitations by adopting multicentred study design to increase sample size, implementing standardized and scheduled radiological imaging protocols independent of rSO_2_ values, and ensuring longer monitoring periods. Such approach would allow for more robust statistical analyses and improve the findings.

In retrospect, the absence of a standardized, scheduled radiological imaging protocol independent of rSO_2_ values represents an important limitation of this study. As noted above, CV may have been missed in four patients who were primarily diagnosed with DCI due to delayed radiological evaluation. It should also be emphasized, however, that delayed cerebral ischemia can occur in the absence of CV.

Another limitation of this study is that we were unable to perform subgroup analyses based on different treatment strategies, although treatment effects were not predefined outcomes of the study. In a recently published study by Feulner et al., administration of magnesium sulphate within the first 24 h after subarachnoid haemorrhage was associated with a lower incidence of CV and DCI [33]. Similarly, a comprehensive meta-analysis by Zheng et al., including 3503 patients, demonstrated a potential benefit of magnesium sulphate in reducing the incidence of CV and DCI [34]. At our institution, early administration of magnesium sulphate is part of routine clinical practice for blood pressure management and prophylaxis against CV and DCI. Nevertheless, it should be noted that the 2023 American Heart Association/American Stroke Association guidelines for the management of aSAH do not recommend the routine use of magnesium sulphate [8].

As discussed above, it is also possible that the predefined rSO_2_ threshold values applied in this study were overly stringent, which may have contributed to the limited sensitivity and specificity of NIRS for the early detection of CV. The use of more permissive cut-off values might allow for improved diagnostic performance in identifying CV in future studies.

It should also be noted that continuous monitoring for up to 7 days was challenging in agitated and excessively perspiring ICU patients, as NIRS sensors frequently became detached. This resulted in intermittent monitoring interruptions and may have led to temporary data loss. In addition, in patients who underwent surgical aneurysm obliteration, rSO_2_ values increased markedly postoperatively, likely due to postoperative subcutaneous edema, suggesting that the measurements may have reflected subcutaneous tissue oxygenation rather than true rSO_2_.

## 5. Conclusions

This study demonstrated limited diagnostic utility of NIRS for the early detection of CV in intensive care patients with a SAH. From a clinical perspective, NIRS should not be considered as a stand-alone diagnostic modality for this purpose. However, as a non-invasive and continuous bedside monitoring technique, NIRS may provide clinically meaningful information when integrated into a multimodal neuromonitoring strategy for neurological intensive care patients. Dynamic trends and abrupt decreases in rSO_2_ may act as early warning signals of cerebral deterioration, prompting timely radiological assessment or escalation of therapy, especially in patients with limited or unreliable neurological examination. Moreover, the association between lower average rSO_2_ values and greater desaturation magnitude with poorer neurological outcomes suggests that NIRS may have a supportive prognostic role in neurological intensive care patient population.

## Figures and Tables

**Table 1:** Study cohort characteristics.

Characteristics	General *n* = 30 (100%)	CV *n* = 6 (20%)	DCI *n* = 7 (23%)	CV + DCI *n* = 4 (14%)	None *n* = 13 (43%)	*p* Value *
**Demographic characteristics:**						
Gender, females *n* (%)	19 (63)	1 (17)	5 (71)	3 (75)	10 (77)	0.07
Age, mean ± SD (yr)	62 ± 12	59 ± 12	68 ± 9	58 ± 12	60 ± 14	0.43
BMI, mean ± SD (kg/m^2^)	27.3 ± 5.8	30.4 ± 7.7	27.5 ± 8.2	25.7 ± 5.4	26.4 ± 3	0.7
**Comorbidities:**						
Hypertension, *n* (%)	24 (80)	5 (83)	6 (86)	4 (100)	9 (70)	0.55
Atherosclerosis, *n* (%)	8 (27)	4 (67)	-	-	4 (31)	0.1
Diabetes mellitus, *n* (%)	1 (3)	1 (17)	-	-	-	NA
Smoking, *n* (%)	12 (40)	1 (17)	4 (57)	1 (25)	6 (46)	0.24
Obesity, *n* (%)	6 (20)	2 (33)	2 (29)	1 (25)	1 (8)	0.43
**SAH characteristics:**						
GCS at admission, median (range)	12 (5–15)	13 (5–14)	12 (5–15)	6 (5–14)	13 (5–15)	0.5
Fisher score grade IV, *n* (%)	25 (83)	6 (100)	5 (71)	4 (100)	10 (77)	0.38
Anterior circulation of aneurysm, *n* (%)	28 (93)	6 (100)	6 (86)	4 (100)	12 (92)	0.38
Therapy **:						
Nimodipine orally, *n* (%)	16 (53)	1 (17)	4 (57)	-	11 (85)	NA
Nimodipine orally + IV, *n* (%)	5 (17)	-	3 (43)	-	2 (15)	NA
Nimodipine orally + IA, *n* (%)	4 (13)	2 (33)	-	2 (50)	-	NA
Nimodipine orally + IV + IA, *n* (%)	5 (17)	3 (50)	-	2 (50)	-	NA
Magnesium sulphate, *n* (%)	24 (80)	5 (83)	7 (100)	3 (75)	9 (69)	NA
Vasoactive drugs, *n* (%)	10 (33)	2 (33)	3 (43)	2 (50)	3 (23)	NA
**Outcomes:**						
mRS at hospital discharge, median (range)	5 (0–5)	5 (3–5)	5 (5)	3 (1–5)	5 (0–5)	0.23
Length of stay in ICU, days (median)	11 (3–43)	14 (3–25)	22 (5–31)	9 (7–28)	6 (4–43)	0.26
Length of hospital stay, days (median)	26 (3–64)	29 (10–41)	28 (5–61)	13 (9–28)	31 (4–64)	0.38
Intrahospital mortality, *n* (%)	9 (30)	2 (33)	3 (43)	2 (50)	2 (15)	0.45

yr—years, BMI—body mass index, GCS—Glasgow Coma scale, mRS—modified Rankin score, ICU—intensive care unit, CV—cerebral vasospasm, DCI—delayed cerebral ischemia, IV—intravenously, IA—intraarterially, and NA—not applicable. * *p* values represent overall comparisons across outcome groups. ** Treatment variables reflect clinical management decisions and are presented descriptively.

**Table 2:** Regional Cerebral Oxygenation and Functional Outcome Assessed by the Modified Rankin Scale.

Modified Rankin Score	rSO_2_, % (Mean ± SD)	Mean rSO_2_ Change from Baseline, % (Mean ± SD)	Maximal rSO_2_ Decrease from BL (%)
0	79 ± 9	+6 ± 13%	−26%
1	69 ± 7	−1 ± 9%	−24%
2	70 ± 8	−0.5 ± 9%	−25%
3	72 ± 9	+4 ± 11%	−21%
5	69 ± 10	−2 ± 19%	−56%
***p*** value *	<0.05	<0.05	

rSO_2_—regional cerebral oxygenation, BL—base line. * *p* values represent overall comparisons across mRS categories.

**Table 3:** Comparison of regional cerebral oxygenation between survivors and non-survivors.

Group	Mean rSO_2_, (Mean ± SD)	rSO_2_ Change from Baseline, % (Min–Max)
Survivors	70 ± 10	−56% to +70%
Non-survivors	72 ± 8	−59% to +35%
***p*** **value**	<0.05	0.137

rSO_2_ change from baseline represents the range of maximal decreases and increases observed within each group.

## Data Availability

The data presented in this study are not publicly available due to ethical and privacy restrictions. De-identified datasets may be provided by the corresponding author upon reasonable request and with permission from the Riga Stradiņš University Ethics Committee and Riga East University Hospital.

## Abbreviations

The following abbreviations are used in this manuscript:

aSAH	Aneurysmal subarachnoid haemorrhage
SAH	Subarachnoid haemorrhage
DCI	Delayed cerebral ischemia
CV	Cerebral vasospasm
NIRS	Near-infrared spectroscopy
rSO_2_	Regional cerebral oxygen saturation
ICU	Intensive care unit
CT	Computed tomography
CTA	Computed tomography angiography
MR	Magnetic resonance
DSA	Digital subtraction angiography
GCS	Glasgow Coma Scale
BMI	Body mass index
mRS	Modified Rankin Scale
INM	Invasive neuromonitoring
TCD	Transcranial Doppler
ROC	Receiver operating characteristic
AUC	Area under the curve
SD	Standard deviation
SPSS	Statistical Package for the Social Sciences
CI	Confidence interval
IV	Intravenously
IA	Intraarterially
COVID-19	Coronavirus disease 2019
AHA	American Heart Association
ASA	American Stroke Association
